# The Effect of the Crucible on the Temperature Distribution for the Growth of a Large Size AlN Single Crystal

**DOI:** 10.3390/ma15010054

**Published:** 2021-12-22

**Authors:** Yue Yu, Botao Liu, Xia Tang, Botao Song, Pengfei Han, Sheng Liu, Bing Gao

**Affiliations:** The Institute of Technological Sciences, Wuhan University, Wuhan 430072, China; 2019106520025@whu.edu.cn (Y.Y.); 2018106520020@whu.edu.cn (B.L.); 2018106520022@whu.edu.cn (X.T.); 2020106520027@whu.edu.cn (B.S.); 2020106520028@whu.edu.cn (P.H.); victor_liu63@vip.126.com (S.L.)

**Keywords:** numerical simulation, thermal design, thermal stress, PVT growth, AlN single crystals

## Abstract

The appropriate distribution of temperature in the growth system is critical for obtaining a large size high quality aluminum nitride (AlN) single crystal by the physical vapor transport (PVT) method. As the crystal size increases, the influence of the crucible on the temperature distribution inside the growth chamber becomes greater. In order to optimize the field of temperature and study the specific effects of various parts of the crucible on the large size AlN single crystal growth system, this study carried out a series of numerical simulations of the temperature field of two crucibles of different materials and put forward the concept of a composite crucible, which combines different materials in the crucible parts. Four composite crucible models were established with different proportions and positions of tantalum carbide (TaC) parts and graphite parts in the crucible. Calculations reveal that different parts of the crucible have different effects on the internal temperature distribution. The axial temperature gradient at the crystal was mainly governed by the crucible wall, whereas the temperature gradient was determined by the integrated effect of the crucible lid and the crucible wall in the radial direction. One type of composite crucible was chosen to minimize the thermal stress in grown AlN crystal, which is applicable to the growth of large sized AlN crystals in the future; it can also be used to grow AlN single crystals at present as well.

## 1. Introduction

As a third-generation semiconductor material, AlN has the advantages of a wide band gap, high resistivity, and high thermal conductivity [1]. Furthermore, both AlN and gallium nitride (GaN) crystals have a wurtzite structure, and their lattice and thermal expansion coefficients are quite close. Thus, compared with sapphire and silicon carbide (SiC), AlN is ideal for III-nitrides epitaxial growth and the ternary compounds [2]. However, at present, due to the lack of ideal III-nitride single crystal substrates, it has severely restricted the improvement of the lifetime and performance of III-nitride lasers and microelectronic devices. Therefore, it is necessary to research and develop the growth technology of AlN bulk single crystals to provide lattice-matched substrate materials for the epitaxial growth of devices and materials. 

Due to the fact that the melting points of AlN are theoretically calculated to be as high as 2800 °C and the dissociation pressure to be 20 MPa, it is challenging to apply the melt Czochralski method to grow an AlN single crystal [3]. An AlN bulk single crystal generally grows at a high temperature via the PVT method [4]. Since the performing experiments are too time-consuming and expensive, numerical simulations allow preliminarily studies of the growth mechanism of an AlN bulk single crystal and the design of optimization schemes for the crystal growth process [5]. Some valuable simulation studies on the growth and material properties of AlN bulk single crystals have been conducted after Slack and Mcnelly [6] reported high purity A1N single crystal growth by the PVT method at an early stage. Dryburgh [7] estimated the maximum possible rate of substance transport and determined the possible rate-limiting steps by simple kinetic theory during sublimation. Inspired by this, Segal et al. [8] formulated the first one-dimensional model considering the convection and diffusion transport, as well as the kinetic constraints on the desorption and adsorption of N_2_. After that, Liu [9] developed an elaborate 2D model containing both Stefan flow and thermal convection. Then, Bogdanov et al. [10] evaluated mass transport in the crucible to learn the species interchange effect upon the aluminum nitride growth rate about the environment and the crucible. The first global model was developed by Liu and Edgar [11] for simulating AlN sublimation growth including surface kinetics. To summarize the research mentioned earlier, Wu and Zhang [12] set out the model of diffusive transport, the model ruled by Al vapor, and the model ruled by N_2_, and the operational terms that allow the implementation of different models were determined. Moreover, Cai et al. [13] accomplished the simulation by developing an internal integrated model to characterize conductive, radiative, and inductive heat transfer. Furthermore, Lee et al. [14] adopted a 3D numerical finite element modeling method to provide a detailed comparison of the distributions of residual interfacial thermal stress induced in AlN crystals deposited upon various substrates. Wolfson [15] investigated the growth rate dependence on the N_2_ pressure. In the last decade, the numerical model has been constantly improved. Gao et al. [16] designed a compressible flow model, which is full-coupled to investigate the mass and sublimation transfer during the AlN crystal growth. Wang et al. [17,18] developed a completely 2D incompressible flow model and a 3D thermoelastic stress model to examine the effects of the crucible configuration on the mass distribution and transport and development of the total resolved shear stress of AlN crystal with numerical experiments.

The above research was all based on the small size AlN single crystal, which is below 50 mm. Since the growth process generates dislocations and defects, it is challenging to grow a larger-size AlN single crystal under the current experimental conditions. A numerical study can overcome this obstacle. The problem is solved and optimized to provide guidance and ideas for subsequent lab tests and commercial production of the AlN single crystal. The crucible is the main component used for crystal growth, and as the crystal size increases, the crucible has an increasing influence on the temperature distribution inside the cavity. Since crystals grown by the PVT method are very sensitive to temperature, and changes in temperature distribution can seriously affect the crystal growth quality, it is also necessary to study the effect of the crucible on the temperature distribution during the growth of large size AlN crystals.

Based on the above, the numerical simulations are conducted to accurately restore the temperature distributions of two crucibles with different materials, graphite crucible and TaC crucible, when growing a 90 mm AlN single crystal. Furthermore, the concept of the composite crucible combining two crucible materials is proposed, and the influence of different crucible parts on the temperature distribution is obtained by changing the proportion of the two crucible materials in the crucibles. Finally, one type of composite crucible that can reduce the internal thermal stress of the AlN crystal while maintaining a regular growth rate is carried out.

## 2. Simulation

**Figure 1:**
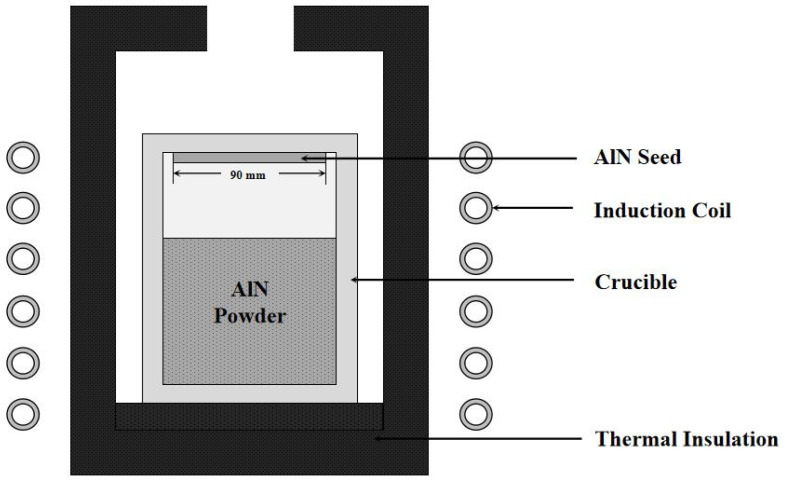
A sketch of the growth cell of AlN crystal.

The basic process of the PVT method for growing AlN crystal can be described as follows: In a high-temperature field, AlN charge powder decomposes and sublimates to produce the gaseous components aluminum vapor (A1) and N_2_ vapor, driven by the negative axial temperature gradient; the gas phase components move to the AlN seed with relatively low temperature, and adsorb, migrate, crystallize, and desorb on the growth interface. This process continues. Then, the growth interface continues to move to the AlN powder region and grows into an AlN bulk crystal. Figure 1 presents a sketch of the growth cell of AlN crystal grown via the PVT method. In addition, the growth chamber is in a nitrogen atmosphere, it is heated by an induction coil (10 kHz), and the crucible needs to be covered by graphite felt to keep the temperature [19,20].

Since the principal objective of this study deals with the optimization and simulation of the temperature field, according to [21], the gas convective heat transfer plays a small role in the temperature field. It can be negligible during the computation. Due to the symmetry of the crystal structure as well as the structure of the growth furnace system, the model can be simplified to a two-dimensional axisymmetric model in a cylindrical coordinate system. The models are analyzed by the finite element method (FEM). Assuming that the growth is in a steady-state equilibrium, the simulation steps can be summarized below: First, the temperature profile of the growth furnace is calculated with an established heat transfer model that considers heat conduction and heat radiation. Then, the first step temperature profile is taken as a boundary condition to calculate the intact model, including thermal stress. 

The heat transfer governing equation is characterized as [22]:

(1) $$\rho C_{p}\frac{\partial T}{\partial t}+\nabla \left(k\nabla T\right)=Q$$

(2) $$\frac{Q_{j}}{\varepsilon_{j}}-\sum_{k=1}^{N}F_{j,k}\frac{1-\varepsilon_{k}}{\varepsilon_{k}}Q_{k}=\sigma T_{j}^{4}-\sum_{k=1}^{N}F_{j,k}\sigma T_{k}^{4}$$

where *ρC_p_* is the effective heat capacity, *T* is the Kelvin temperature, *t* is the time, *k* is the thermal conductivity, *Q* is the radiative heat flux, *ɛ_j_, ɛ_k_* is the emissivity, *F*_*j*,*k*_ is the view factor, and *σ* is the Stefan Boltzmann constant.

The thermal physical and material properties of the system components are shown in Table 1 [23].

Since AlN crystal growth is in a nonequilibrium state, thermal stress is induced within the crystal throughout the growth process, which is the main driving force for the creation, slip, and proliferation of dislocations in the AlN crystal [24].

Assuming that the material is a linear elastic body, the governing equations of the thermal stress field are [25]:

(3) $$\frac{1}{r}\frac{\partial }{\partial r}\left(r\sigma_{rr}\right)+\frac{\partial \tau_{rz}}{\partial z}-\frac{\sigma_{\varphi \varphi }}{r}=0$$

(4) $$\frac{1}{r}\frac{\partial }{\partial r}\left(r\tau_{rz}\right)+\frac{\partial \sigma_{zz}}{\partial z}=0$$

where *σ_rr_*, *σ_φφ_* and *σ_zz_* denote the normal stresses; *τ_rz_* denotes the shear stress.

In addition, according to Hooke’s law of thermoelastic solids, AlN crystal is a thermoelastic anisotropic body, and the stress–strain relation of AlN crystal can be taken as [26]:

(5) $$\left(\begin{array}{l} \sigma_{rr}\\ \sigma_{\varphi \varphi }\\ \sigma_{zz}\\ \tau_{rz} \end{array}\right)=\left(\begin{array}{cccc} c_{11} & c_{12} & c_{13} & 0\\ c_{12} & c_{22} & c_{23} & 0\\ c_{13} & c_{23} & c_{33} & 0\\ 0 & 0 & 0 & c_{44} \end{array}\right)\times \left(\begin{array}{c} \varepsilon_{rr}-\alpha_{r}\left(T-T_{ref}\right)\\ \varepsilon_{\varphi \varphi }-\alpha_{\varphi }\left(T-T_{ref}\right)\\ \varepsilon_{zz}-\alpha_{z}\left(T-T_{ref}\right)\\ \varepsilon_{rz} \end{array}\right)$$

where *c_ij_* represents the elastic constant, *ε_rr_*, *ε_φφ_*, *ε_zz_*, *ε_rz_* represent the strain components, *α_r_*, *α_φ_*, *α_z_* represent the thermal expansion coefficients, and due to the hexagonal structure, the thermal expansion coefficient of AlN crystal has only two independent components, *α _r_*= *α_φ_*. *T_ref_* represents the reference temperature, which is chosen as the lowest temperature in the AlN seed.

The strain components can be described as [27]:

(6) $$\begin{array}{l} \varepsilon_{rr}=\frac{\partial u}{\partial r}\\ \varepsilon_{\phi \phi }=\frac{u}{r}\\ \varepsilon_{zz}=\frac{\partial w}{\partial z}\\ \varepsilon_{rz}=\frac{\partial u}{\partial z}+\frac{\partial w}{\partial r} \end{array}$$

where *u* and *w* represent the horizontal and vertical displacements, respectively.

## 3. Results and Discussions

### 3.1. Effect of Crucible Material on Temperature Distribution

**Figure 2:**
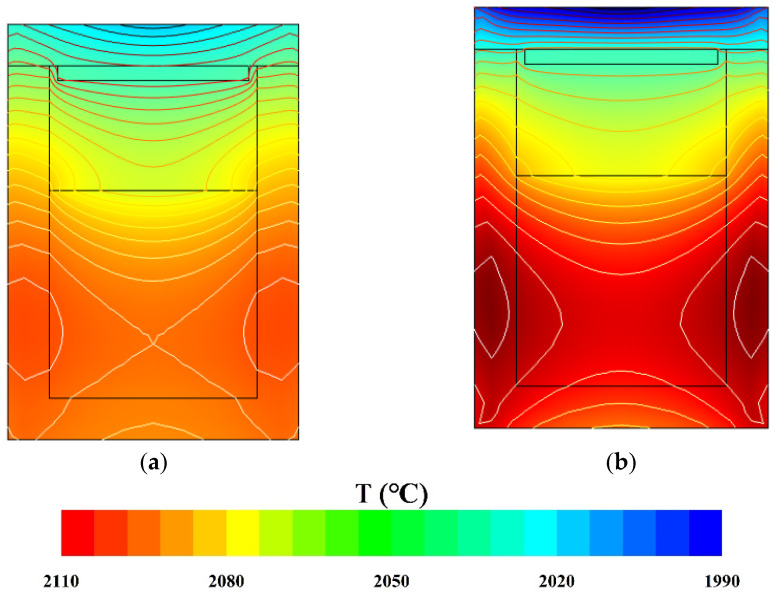
Distributions of temperature in (**a**) Graphite crucible and (**b**) TaC crucible.

Two growth systems were set up in simulation with a difference in crucible materials. According to the literature [28,29], the two most commonly used crucible materials for AlN growth by PVT are high-purity tungsten (W) metal and tantalum carbide, which are selected based on the fact that the two crucible materials do not interact with each other and do not have the same heat transfer capacity. Since W reacts with TaC at high temperatures to form WC, which affects the quality and lifetime of the composite crucible, graphite and TaC were chosen to meet both conditions and were easy to prepare. The temperature distributions of the two crucibles under the same heating conditions are displayed in Figure 2. In general, the temperature distributions of both crucibles were quite similar. The temperature distribution in the crystal growth region exhibited a trend of the lower high and upper low. Thus, a negative temperature gradient formed axially in the gas area between the AlN crystal and the powder source (the growth region). It was the major driving force of the AlN crystal growth. In contrast, at the bottom part, the temperature distribution in the graphite crucible was gentler than that in the TaC crucible. At the top part, it was the exact opposite condition. The influence of the crucible material on the temperature distribution was different in various parts. This difference was mainly related to the thermal conductivity of the crucible material itself. The higher the thermal conductivity inside the material, the higher the temperature gradient will be.

During the simulation of AlN crystal growth, the influence of impurities was ignored. The crucible mainly contains two vapor species, Al and N_2_. The crystal growth rate *V_g_* is deduced from the growth kinetics, which is expressed as follows [30]:

(7) $$V_{g}=k_{g}\frac{\exp\left(A-\frac{B}{T}\right)}{P^{1.5}T^{1.2}}\frac{\Delta T}{z}$$

where *k_g_* is the growth rate coefficient obtained from the experiment, *k_g_* = 407.539, *A* and *B* are the constants based on the thermodynamic data, *A* = 27.055, *B* = 75788, *P* is the internal pressure of the furnace, and Δ*T* and *z* are the difference of temperature and the length between the AlN powder and AlN seed, respectively.

As the gas convective is neglected in the calculation, the pressure *P* is regarded as a fixed value in this research, *P* = 300 Torr. According to Equation (8), only the temperature distribution dictates the growth rate. We set the center of the crystal surface as the temperature control point. Then the growth rate of the AlN crystal was mainly related to the axial gradient of temperature in the growth region.

**Figure 3:**
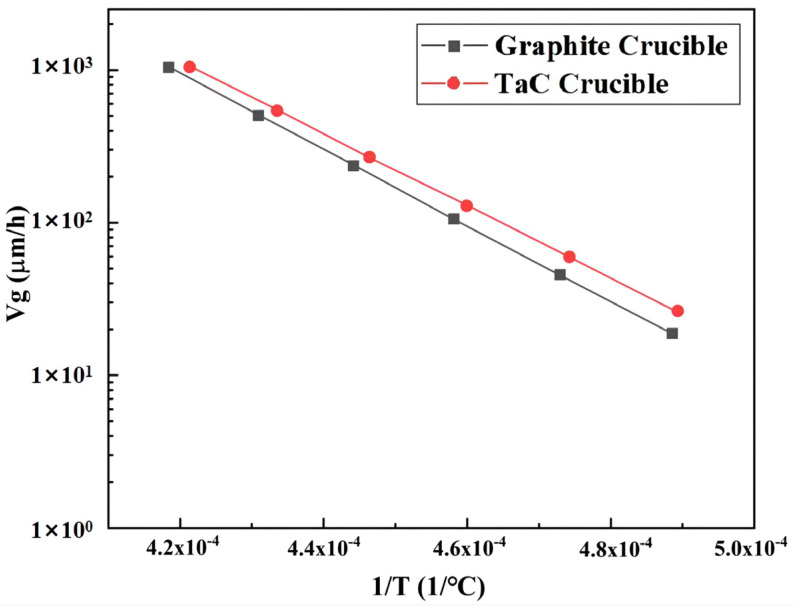
Growth rates of the AlN crystal grown in different crucibles.

Figure 3 illustrates the growth rates of AlN crystals grown in the two crucibles. The growth rate was linearly related to the reciprocal of the temperature. The profiles reveal that AlN crystal grew faster in the TaC crucibles than in the graphite crucibles. From Figure 2, the formation of a higher temperature gradient is evident on the longitudinal wall of the TaC crucible. So, the growth rate of the AlN crystal grown in it is higher than that of the graphite crucible. 

For the purpose of increasing the diameter of the epitaxial growth crystals, it is essential to establish a reasonable temperature distribution of the as grown AlN crystal in the radial direction as well. An excessively large radial temperature gradient causes anisotropic thermoelastic stress of the crystal, resulting in the formation and propagation of various defects, such as low-angle grain boundaries (LAGB), basal plane dislocations (BPD), and so on. The multiple defects formulated during growth are the dominant limitations to AlN single crystal growth. Therefore, to successfully grow a 90 mm aluminum nitride single crystal, the thermal stress generated within it should be minimized.

Current theory believes that the resolved shear stress (RSS) due to the inhomogeneous radial temperature distribution is the main driving force for creating, slipping, and proliferation of the dislocations in the grown AlN single crystal. As a reference, the Von Mises stress (VMS) can be applied to evaluate the level of the stress inside the crystal [31].

(8) $$\sigma_{\mathrm{Mises}}=\sqrt{\frac{1}{2}\left[{\left(\sigma_{zz}-\sigma_{rr}\right)}^{2}+{\left(\sigma_{zz}-\sigma_{\varphi \varphi }\right)}^{2}+{\left(\sigma_{\varphi \varphi }-\sigma_{rr}\right)}^{2}+6\tau_{rz}\right]}$$

When the VMS stress of the AlN crystal excels the critical resolved shear stress (CRSS), dislocations or even cracks occur. Therefore, reducing the VMS stress is an effective method to reduce the risk of dislocation defect formation minimizing the density of dislocations as well as the macro cracks in AlN crystal. 

It is assumed that the crystal surface attached to the crucible lid is rigid, while the other two surfaces of the crystal are not in contact with the crucible wall and stress-free. The boundary conditions for thermal stress calculations can be expressed as:

(9) $$\begin{array}{ll} u=0,v=0 & \mathrm{at}\;z=0\\ u=0,\frac{\partial v}{\partial r}=0 & \mathrm{at}\;r=0\\ \sigma \cdot n=0 & \mathrm{at}\;\mathrm{the}\;\mathrm{two}\;\mathrm{free}\;\mathrm{moving}\;\mathrm{surfaces} \end{array}$$

**Figure 4:**
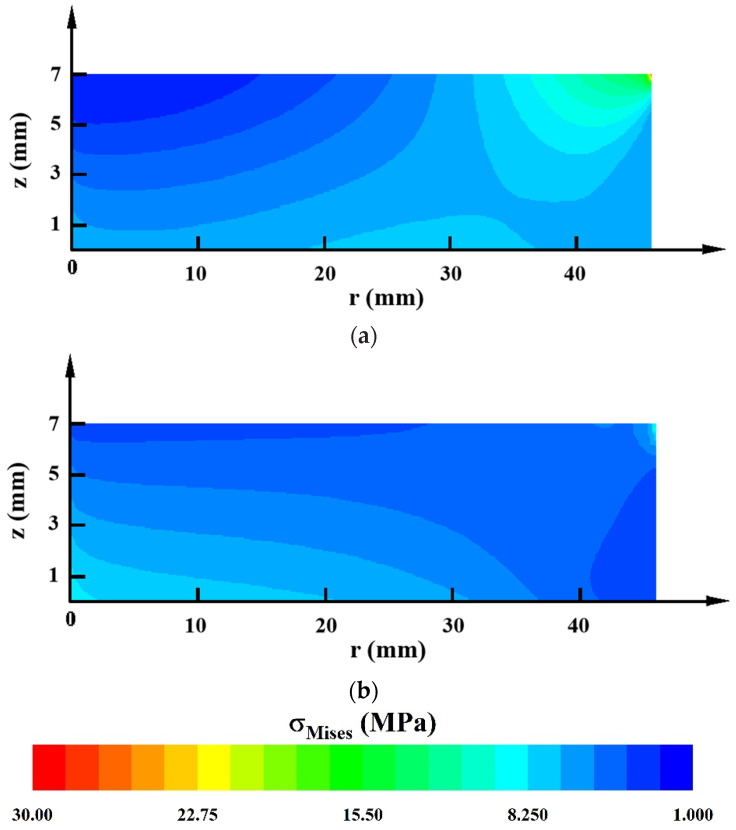
Distributions of thermal stress inside the AlN crystals grown in (**a**) Graphite crucible and (**b**) TaC crucible.

The Von Mises stress distributions inside the grown AlN crystals in the two crucibles are shown in Figure 4. The maximum stress appeared at the crystal top edge on account of the rigid body. The minimum stress occurred at the center of the crystal surface attached to the crucible lid, where the temperature was the lowest, and the stress distributions near the crystal surfaces tended to be flat. The stress level of the AlN crystal grown in the graphite crucible was relatively high. As for the TaC crucible, since the internal radial temperature distribution is more uniform, the influence of the rigid body constraint was reduced. So, the stress of the fixed AlN crystal grown inside it is smaller. In addition, the high temperature at the free surface of the crystal led to higher stress there.

From the above calculations, the high thermal resistance of the TaC crucible allows for more uniform temperature distribution in the radial direction, which results in lower thermal stress.

The difficulty in growing a large-sized AlN single crystal lies in the huge thermal stresses generated during the lateral growth of the crystal. As a result, the crystal defects and dislocations increase, which leads to failure growth. Judging by the calculation of this study, when a material with low thermal conductivity is used as a crucible, the crystal growth rate is high and the growth quality is high, so there is a greater potential to grow large size crystals.

### 3.2. Effect of Crucible Parts on Temperature Distribution 

**Figure 5:**
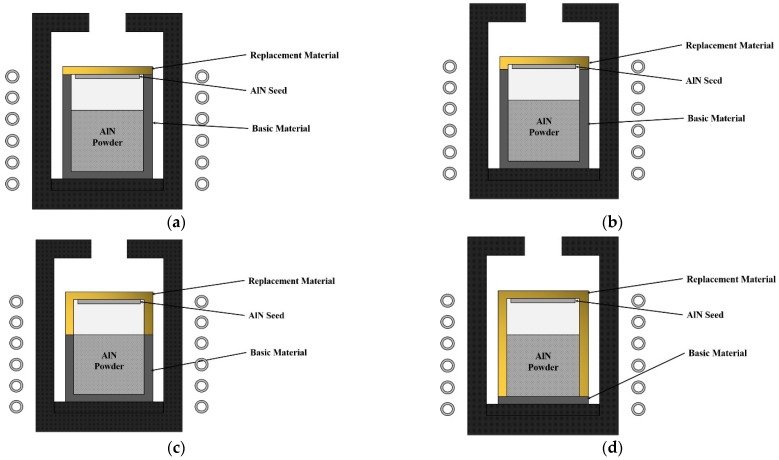
Schematic diagrams of the composite crucibles (**a**) Composite crucible A, (**b**) Composite crucible B, (**c**) Composite crucible C, (**d**) Composite crucible D.

During the calculation, it was found that the various crucible parts had different effects on the distribution of temperature inside the crucible. Therefore, as shown in Figure 5, the above two materials (graphite and TaC) were combined into composite crucibles to determine the impact of the different crucible components on the temperature field. The main difference between these four crucibles is the different proportions of TaC and graphite forming the crucible. It can be seen as replacements of parts of the graphite crucible by TaC, and the replacements change from top to bottom. The details are explained in the description of Figure 5. As this study targets optimizing the growth conditions for large sized AlN single crystal, the dimensions follow the previously designed growth chambers for growing 90 mm AlN single crystal.

**Figure 6:**
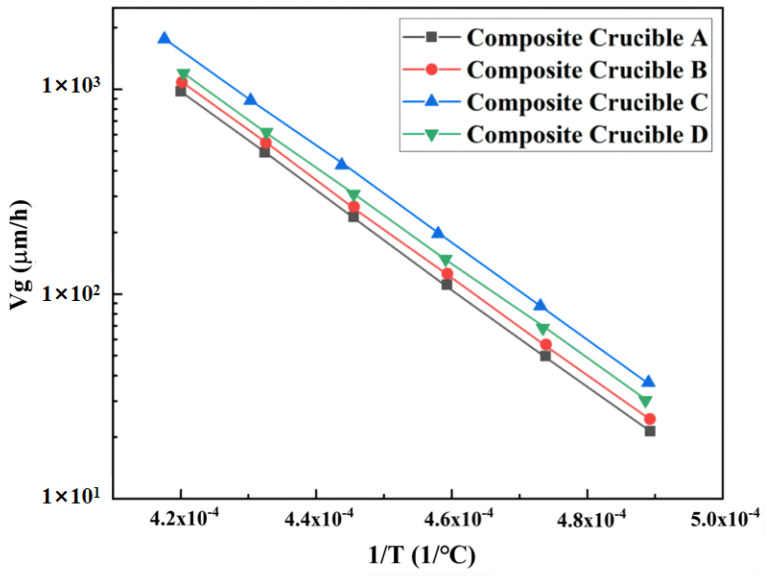
Comparison of the growth rates of the AlN crystals grown in four composite crucibles.

Figure 6 shows the growth rates of the AlN crystals grown in four composite crucibles. Compared with the monomaterial crucibles, the growth rates of the four composite crucibles were higher, of which composite crucible C had the highest growth rate and A the lowest. As mentioned above, the crystal growth rate in this study was mainly affected by the axial temperature gradient. From a partial view, when the crucible parts were of varying thermal conductivity, the stronger thermally conductive part had a higher heat flux. Therefore, the growth rates of AlN crystals in the composite crucibles were higher than in the graphite crucible. On the whole, the combination of two materials with different thermal conductivity generated additional thermal resistance on the contact surface and increased the temperature difference, so that the growth rates were higher than that of the pure TaC crucible also.

Because the longitudinal length of TaC in the composite crucible A and B occupied a very low proportion of the entire crucible, the axial temperature gradients in those two crucibles were lower. However, the gas area of the crucible is the main region for crystal growth, so variations in crucible material in this part significantly affected either the axial temperature distribution or the growth rate. Therefore, the growth rate of crucible C was the largest among the four crucibles. As for the composite crucible D, only the material of the crucible bottom is graphite, which has higher thermal conductivity, so its heat transfer effect was worse than that of C, resulting in a relatively low temperature gradient in the axial direction. 

**Figure 7:**
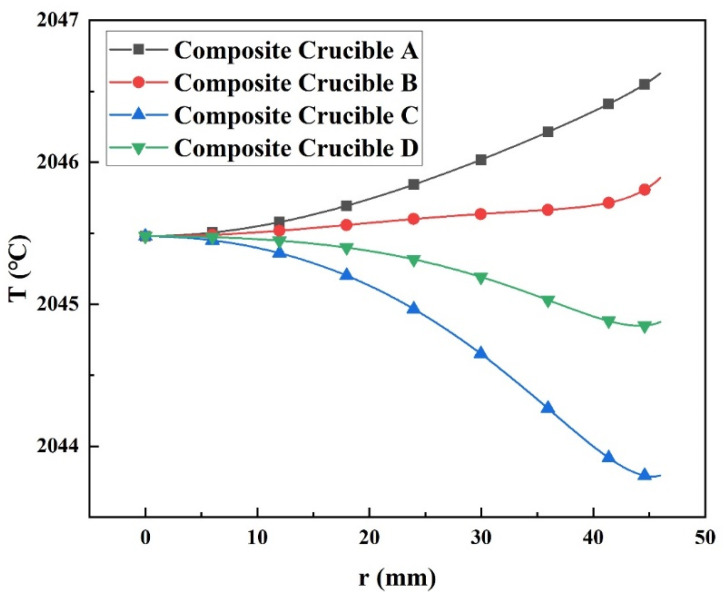
Radial temperature distributions of the AlN crystal horizontal free surface grown in four composite crucibles.

Figure 7 shows the radial temperature distributions of the horizontal free surfaces in the four composite crucibles. The radial temperature gradient turned from positive to negative as the proportion of TaC in the crucibles increased. However, when the TaC crucible part exceeded the AlN powder region, the negative temperature distribution tended to become flat again. It implies that the temperature distribution in the radial direction was not only dependent on the crucible lid, but also on the axial part of the crucible. The radial temperature profile on the surface of crystal grown in the composite crucible B was affected most, which was even flatter than that of the TaC crucible. It indicates that the crystal and its vicinity are the main components that govern the temperature distribution of the AlN crystal in the radial direction.

**Figure 8:**
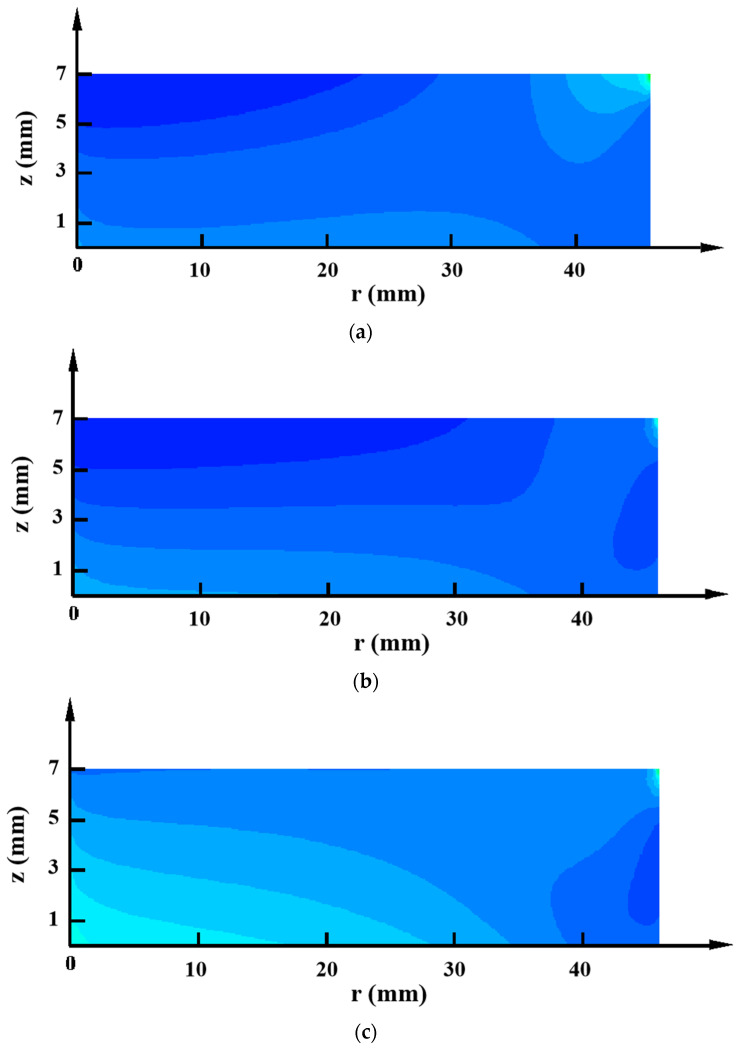

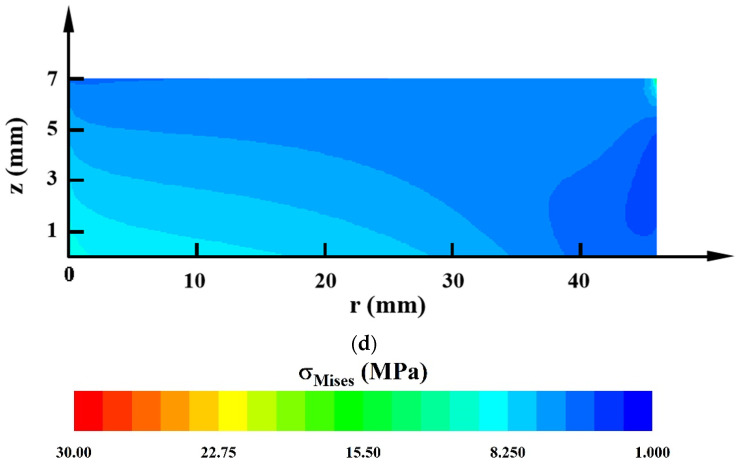
Distributions of thermal stress inside the AlN crystal grown in (**a**) Composite crucible A, (**b**) Composite crucible B, (**c**) Composite crucible C, and (**d**) Composite crucible D.

As Figure 8 shows, the AlN crystal grown in the composite crucible A had a thermal stress distribution resembling that of the W crucible but with a lower stress level. The thermal stress distributions of the crystals in the other three composite crucibles were similar to that of the TaC crucible. Among them, only the thermal stress level of the crystal in the composite crucible B was lower than that of the TaC crucible. Moreover, it is worth noting that the positive and negative radial temperature distributions had a remarkable impact on the level of thermal stress inside the crystals, and the negative radial temperature distribution produced a much higher minimum thermal stress. This was mainly attributed to a shift in the location where the minimum temperature occured. The implication is that a negative temperature gradient of the radial direction should be avoided to minimize the thermal stress generated in the as grown AlN single crystal.

The above discussion reveals that different crucible parts seriously impact the temperature distribution within the growth chamber. The crucible wall significantly influences the axial temperature distribution, while the joint action of the crucible lid and the crucible wall affects the radial temperature distribution. Since the bottom of the crucible is far from the growth region, it has less influence on the temperature distribution within the growth region. Moreover, for the current as well as the future growth of AlN single crystal, the composite crucible formed by the crucible parts covering the crystal region with low thermal conductivity materials and the rest of the high thermal conductivity crucible part has excellent potential for the growth of larger size and higher quality AlN single crystal given the extremely low internal thermal stress in the crystal. 

## 4. Conclusions

Concerning the problem of growing an AlN single crystal with a larger size and higher quality in the future, this paper used a numerical simulation to compare the two most suitable materials for growing 90 mm AlN single crystal concerning thermophysical properties and analyzed the influence of different crucible materials on the temperature field in the AlN crystal growth cell via the PVT method. The results showed that materials with weak thermal conductivity were more suitable as crucibles for growing large size and high quality AlN crystals because of the rapid crystal growth rate as well as the lower thermal stress level of the grown crystal. Moreover, the concept of composite crucible combining graphite and TaC to examine the effect of various crucible parts on the temperature distribution of the AlN crystal growth cell was put forward. According to the result, the temperature distribution in the axial direction was strongly influenced by the crucible wall. In contrast, the temperature distribution in the radial direction was mainly influenced by the joint action of the crucible lid and the crucible wall. The composite crucible formed by the TaC part covering the crystal region and the remaining part graphite minimized the thermal stress in the AlN crystal while ensuring a high growth rate. This study also suggests that a negative radial temperature gradient is not conducive to reducing the thermal stress inside the crystal. It provides a new direction for optimizing the temperature field for growing large-size AlN single crystal in the future.

## Figures and Tables

**Table 1:** Physical parameters of the materials in the growth chamber for simulation.

Material	Thermal Conductivity (W/m·K)	Density (kg/m^3^)	Heat Capacity (J/kg·K)
Graphite Crucible	115	1760	720
TaC Crucible	22	14,300	60.65
Insulation	0.5	170	2100
AlN powder	22.55	27, 0.34	11, 72.7
AlN seed	320	3250	1197

## Data Availability

Not applicable.
